# Characterizing food environments of hospitals and long-term care facilities in the Netherlands: a mixed methods approach

**DOI:** 10.1186/s12913-023-10399-6

**Published:** 2024-01-04

**Authors:** Joline J. Wierda, Emely de Vet, Ellemijn Troost, Maartje P. Poelman

**Affiliations:** Chair group Consumption and Healthy Lifestyles, Wageningen University & Research, Wageningen, The Netherlands

**Keywords:** Food environments, Healthy diets, Hospitals, Long-term care facilities, Healthcare, Patients, Nurses

## Abstract

**Background:**

Hospitals and long-term care facilities, which are key institutions to serve health and well-being, have an important exemplary role in providing supportive food environments to encourage healthy and sustainable food choices. The objective of this study is to characterize the physical, socio-cultural, political and economic dimensions of the food environment for health care receivers, health workforce and visitors in healthcare settings, and make comparisons between the food environment of hospitals and long-term care facilities.

**Methods:**

To characterize the food environment in healthcare settings, two sub-studies were conducted. In sub-study 1, semi-structured interviews were held with staff members (n = 46) representing 11 hospitals and 26 long-term care facilities (rehabilitation centres, nursing homes, institutions for people with intellectual disabilities and mental healthcare institutions). In sub-study 2, staff members audited the food environment in hospitals (n = 28) and long-term care facilities (n = 36) using a predefined checklist.

**Results:**

The food environment in Dutch healthcare settings varies substantially between locations although noticeable differences between hospitals and long-term care facilities were identified. Hospitals and larger long-term care facilities featured more often restaurants and utilized central spaces for preparation of meals, while smaller long-term care facilities often operated as household-like settings. Type of healthcare shaped the socio-cultural food environment, with hospitals primarily emphasizing nutrition for fast recovery, while long-term care facilities more often as an instrument (i.e., to structure the day). Participants highlighted the importance of food policies and broad organizational support for realizing and regulating improvement of the food environment. Yet, long-term care facilities were less familiar with national guidelines for food environments compared to hospitals. Several economical aspects, like profit motives, strict budgets and contracts with external parties affected and shaped the food available within all healthcare settings.

**Conclusions:**

This study characterized the food environment in Dutch healthcare settings. Disclosed differences between hospitals and long-term care facilities should be incorporated in strategies for a transition of the food environment. Future research should investigate the underlying mechanisms of the healthcare food environment attaining all healthcare stakeholders - health care receivers, staff and visitors - while prioritizing sustainability alongside healthiness.

**Supplementary Information:**

The online version contains supplementary material available at 10.1186/s12913-023-10399-6.

## Background

Healthcare organizations, including hospitals and long-term care facilities, are essential environments to serve health and well-being. These organizations have an important role to lead by example in promoting health and sustainability. Healthy diets are key in promoting health, including the prevention of malnutrition and diseases, appropriate healing, recovery and promotion of quality of life and a healthy lifestyle [1, 2]. While a myriad of factors shape peoples’ diet, it is well understood that food environments play a crucial role in shaping food choices and thus health and environmental outcomes [3, 4].

The food environment can be dissected into four dimensions, a physical-, socio-cultural-, political-, and economic dimension, rooted in the analysis grid for environments linked to obesity (ANGELO) framework of Swinburn et al. [5, 6]. The physical dimension of the food environment refers to the food available, its characteristics (e.g. healthiness, quality) and information about the food (e.g. communicated via nutrition labels). The socio-cultural dimension defines the culture, ethos or climate related to food consumption in a particular context (e.g. within a healthcare setting), and includes attitudes, beliefs and values. The political dimension comprises rules, for example food policies or -regulations, laws and standards, for example about food availability within a certain context. Finally, the economic dimension refers to food costs, for example for catering and retailing, but also pricing policies (e.g. taxes) and incentives (e.g. subsidies). In hospitals and long-term care facilities the food environment is characterized by its diversity and the complex interplay of these dimensions, involving multiple stakeholders with diverse interests. Moreover, the food environment has to serve the needs of health care receivers as well as staff and visitors [7]. Although prior studies have explored aspects of the healthcare food environment, there remains a need for a comprehensive understanding of all of its dimensions in order to identify potential areas for intervention and achieve healthy and sustainable food environments in healthcare settings. Such knowledge can identify targets for actions to improve food environments in the healthcare setting, and expose health care receivers, staff and visitors to a healthy food environment, thereby enhancing public and environmental health. The current literature exhibits a scarcity of research focused on this wide perspective, revealing three primary gaps that require further exploration.

First, prior studies predominantly focus on the physical food environment. A recent systematic review into the availability of healthy food and drinks in hospitals in the United Kingdom and United States of America concluded that the nutritional quality of items varies and differs between and within healthcare facilities [8]. Horton Dias et al. [9] found that the consumer food environment in hospitals did not promote a healthy diet, based on observations in cafeterias, vending machines and gift shops in 31 hospitals in the United States of America. Also the food assortment of food outlets in hospitals are predominantly unhealthy and widely available [10–13]. While these studies offer valuable insights, there is still a lack of research on the economic, political and socio-cultural dimensions, leaving important aspects of the healthcare food environment understudied.

Second, to the best of our knowledge, insights into the healthcare food environment predominantly centre around hospitals, leaving out understanding about the food environment of long-term care facilities. However, the healthcare landscape extends far beyond hospitals. Long-term care facilities are equally important as hospitals in promoting health and sustainability. Health care receivers frequently reside in long-term care facilities for longer periods compared to hospital stays, making the food environment there more influential in shaping dietary patterns of health care receivers. The ESPEN guidelines on hospital nutrition do include rehabilitation centres and nursing homes, however, it was indicated that more knowledge is needed for organization of nutritional issues and good patient safety in nutritional care [14]. This has also been acknowledged by the National Prevention Agreement in the Netherlands, an agreement signed in 2018 by the Dutch government and seventy public and private organizations aimed to achieve a healthier Netherlands by reducing and preventing overweight and obesity, smoking and alcohol consumption [15]. Several goals focus on creating healthy food environments and one emphasizes that *by 2025, 50% of hospitals are expected to offer healthy foods to patients, visitors and staff, with the goal of reaching full implementation across all hospitals no later than 2030*. The Nutrition & Healthcare Alliance (the national expertise center dedicated to achieving health benefits through the application of scientific findings on nutrition and exercise in prevention and healthcare), supports to realize this ambition through the national ‘Goede Zorg Proef Je’ program (translated to English: ‘A Taste of Excellent Healthcare’) [16]. By means of ‘Goede Zorg Proef Je’, the Alliance collaborates with several parties including the Dutch Hospital Association (NVZ), Netherlands Nutrition Centre, Dutch Association of Dietitians and private parties (like caterers and food suppliers). Currently, 80% of Dutch hospitals are actively pursuing this ambition with the support of the Nutrition & Healthcare Alliance. Long-term care facilities are also getting involved in these ambitions, but vary substantially in the organization and type of care they deliver. It is therefore currently unknown if the approach for realizing a healthy food environment in hospitals is applicable to long-term care facilities. Comparing these two can provide valuable insights for designing and implementing actions to enhance the food environment in all healthcare settings.

Third and final, characterizing the food environment of healthcare settings is predominantly targeted at the publicly available food options (e.g., for everybody) or staff restaurants, with little regard for the food environment of inpatients or health care receivers. For example, one study found that hospitals nurses experiences the food environment in hospitals oppressively unhealthy [17] and others concluded that health care staff heavily favoured healthy foods [18]. Another study reported that visitors of a hospital percepted low availability of healthy food options [19]. And Lederer et al. [20] described that supporting a healthy food environment had no priority for staff managing cafeterias in hospitals. Moreover, the priorly mentioned review of Richardson et al. [8] fully excluded the food environment for inpatients.

This study will address the three aforementioned gaps and will add to the literature a complete picture of the totality of the food environment in the healthcare setting. The objective of this study is to characterize the food environment in the healthcare setting in the Netherlands and compare the food environment between hospitals and long-term care facilities, both specifically concerning health aspects of the food environment.

## Methods

This study used a mixed methods approach to assess the food environment, divided into sub study 1 (qualitative approach) and sub study 2 (quantitative approach). The study was approved by the Social Sciences Ethics Committee of Wageningen University & Research and it complies with the Netherlands Code of Conduct for Research Integrity. The study was part of a project that was financially supported by a grant (grant number 162135) from the Regio Deal Foodvalley, a collaboration between the Dutch government and different regional governments, entrepreneurs, education- and knowledge institutions, including the Nutrition & Healthcare Alliance.

The sampling frame for both sub study 1 and sub study 2 included all intramural healthcare facilities in the Netherlands where health care receivers reside including hospitals and long-term care facilities (nursing homes, rehabilitation centres, institutions for people with intellectual disabilities and institutions for mental healthcare). Exclusion criteria were extramural healthcare facilities or polyclinical care institutions. Via the network of the Nutrition & Healthcare Alliance and several healthcare associations in the Netherlands, both convenience and purposive sampling were used to recruit hospitals and long-term care facilities. Then, via existing key-contacts or general email addresses of the organizations, participants for both sub study 1 and sub study 2 were invited when they were professionally engaged with the food environment within their healthcare organization (e.g., facility manager, dietitian, food service manager or similar). Participants in sub study 1 do not necessarily correspond with those in sub study 2. The emphasis during this study was on health and less on sustainability, however when following the national dietary guidelines the consumption pattern is generally also more sustainable.

### Sub study 1

#### Design

Semi-structured interviews were conducted with staff of hospitals and long-term care facilities to assess four (physical, socio-cultural, political and economic) dimensions of the food environment.

#### Sample and participant characteristics

A total of 37 interviews were conducted with 46 participants, of which 29 individual interviews, 7 interviews with 2 participants and one interview with 3 participants. The interviewees represented 11 hospitals, 6 nursing homes, 6 rehabilitation centers, 5 institutions for people with intellectual disabilities and 9 mental healthcare institutions. General characteristics of the interview participants can be found in Table 1.

**Table 1:** Participant characteristics of sub-study 1 (qualitative semi-structured interviews)

Participant #	Representing hospital or long-term care setting	Gender	Function of participant	Individual, two or three participants during interview
P1	Hospital #1	Female	Head of hotel services	Individual (live)
P2	Hospital #2	Male	Team leader catering	Individual (online)
P3	Hospital #3	Female	Policy advisor food and beverages	Individual (live)
P4	Hospital #4	Male	Manager hotel services	Individual (online)
P5	Hospital #5	Female	Project leader catering and services	Individual (online)
P6	Hospital #6	Male	Catering coordinator	Two (live)
P7	Hospital #6	Female	Manager foodservice & hospitality	Two (live)
P8	Hospital #7	Female	Leader nutrition program	Individual (online)
P9	Hospital #8	Female	Facilities manager	Two (online)
P10	Hospital #8	Female	Head of dietitian department	Two (online)
P11	Hospital #9	Female	Head of nutrition department	Individual (online)
P12	Hospital #10	Male	Implementation coordinator inpatient catering	Three (online)
P13	Hospital #10	Female	Coordinator nutrition and quality	Three (online)
P14	Hospital #10	Male	Team leader staff catering	Three (online)
P15	Hospital #11	Female	Manager hotel services	Individual (online)
P16	Intellectual disabilities #1	Male	Hospitality manager	Individual (online)
P17	Intellectual disabilities #2	Female	Team leader client services	Individual (online)
P18	Intellectual disabilities #3	Female	Assistant living	Two (online)
P19	Intellectual disabilities #3	Female	Assistant living	Two (online)
P20	Intellectual disabilities #4	Male	Director	Individual (online)
P21	Intellectual disabilities #5	Female	Team leader specialistic long-term care	Two (online)
P22	Intellectual disabilities #5	Female	Care assistant	Two (online)
P23	Rehabilitation #1	Female	Head of residing services	Individual (online)
P24	Rehabilitation #2	Female	Manager housing, services and facilities	Individual (online)
P25	Rehabilitation #3	Male	Team manager business operations	Individual (online)
P26	Rehabilitation #4	Female	Facilities manager	Individual (online)
P27	Rehabilitation #5	Male	Nutrition manager	Individual (online)
P28	Rehabilitation #6	Female	Dietitian	Individual (online)
P29	Mental health #1	Male	Psychiatric nurse	Individual (online)
P30	Mental health #2	Female	Psychiatric nurse	Individual (online)
P31	Mental health #3	Male	Facilities manager	Individual (online)
P32	Mental health #4	Female	Chef	Two (online)
P33	Mental health #4	Male	Chef	Two (online)
P34	Mental health #5	Male	Head of facility services	Two (live)
P35	Mental health #5	Male	Concierge	Two (live)
P36	Mental health #6	Male	Team leader food and beverages	Individual (online)
P37	Mental health #7	Female	Nurse	Individual (online)
P38	Mental health #8	Male	Coordinator services	Individual (online)
P39	Mental health #9	Female	Practice assistant	Two (live)
P40	Mental health #9	Female	Assistant living	Two (live)
P41	Nursing home #1	Female	Team manager food and beverages	Individual (online)
P42	Nursing home #2	Female	Manager transition facilities services	Individual (online)
P43	Nursing home #3	Female	Ad interim facilities manager	Individual (online)
P44	Nursing home #4	Male	Director	Individual (online)
P45	Nursing home #5	Male	Facilities manager	Individual (online)
P46	Nursing home #6	Female	Chef	Individual (live)

#### Procedure

Interviews were conducted between July 2021 and February 2022 and the majority was administered online (n = 31) via Microsoft Teams and a minority face-to-face (n = 6). Participants were invited via e-mail, received an information letter with explanation and purpose of the interview and all provided signed informed consent. The principle of saturation was applied for each type of healthcare institution to determine the sample size. The interviews with hospitals and institutions for intellectual disabilities were conducted by one author (JJW) (n = 16) and the interviews with nursing homes, mental healthcare institutions and rehabilitation centers were conducted by another author (ET) (n = 21). Interview duration ranged from roughly 40 to 90 min. Interviews were audio-recorded and were transcribed verbatim by one of the authors (ET) or by an external company (Transcript Online) and anonymized.

#### Interview guide

The interviews were semi-structured to allow room for emerging concepts. An interview guide was created for this study and pilot tested within one hospital and minor adjustments were made in e.g. the order of questions. The interview guide was used to obtain information regarding four dimensions of the food environment in hospitals and healthcare institutions, see Table 2 for a concise version of the interview guide with exemplary questions. The full interview guide (translated from Dutch to English) can be found in Additional file 1.

**Table 2:** Concise version interview guide with exemplary questions

Topics	Prompts
Physical dimension food environment: organisation, facilities	How are the food and drinks organised, for health care receivers, staff and visitors? Which facilities are in place?
Social cultural dimension food environment: attitude, culture, modelling, empowerment	How do health care receivers, staff, management board in the healthcare organization think about healthy and sustainable food and drinks? What are the norms, values, traditions concerning healthy and sustainable food and drinks? Nutritional needs health care receivers per type of care. Exemplary and modelling role of organization and staff. Empowerment of health care receivers, staff, visitors and external parties, e.g. caterers.
Political dimension food environment: policy, rules, guidelines	Having a policy on food within the healthcare organization, or reason why not, content of the policy for health care receivers, visitors, staff, policy created by whom, specific content on healthy and sustainable food and drinks, use of guidelines, restrictions.
Economic dimension food environment: profit and loss, price, in-house/outsourced, promotion	Economic considerations to sell/buy food and drinks and differences per facility, promotion of food and drinks, price for food and drinks for health care receivers, visitors, staff

#### Data analysis

First, two authors (ET and JJW) read through two different transcripts independently, discussed impressions, built consensus and created a codebook. Starting with a deductive approach, including codes based on the interview guide, followed by an inductive approach as new codes emerged from the transcripts and were included in the coding frame. Second, two authors (ET and JJW) independently coded each half of all the transcripts with the codebook using ATLAS.ti Windows (Version 9.1). The codes were grouped into main themes by both authors (ET and JJW), the four dimensions of the food environment and the process of thematic analysis was used to report the results. The four dimensions of the food environment were explored in the light of the distinction between hospitals and long-term care facilities and among health care receivers, staff and visitors. The results were illustrated with quotes derived from the interviews and translated from Dutch to English.

### Sub study 2

#### Design

Sub study 2 used a cross-sectional observational design, where staff of hospitals and long-term care facilities audited the food environment within their organization with a digital inventory checklist.

#### Recruitment and procedure

Stakeholders were invited via email to participate in sub study 2 between November 2021 and March 2022. These stakeholders received the purpose and explanation of the study and an online link for the checklist (using Qualtrics software (Qualtrics, Provo, UT). Reminder emails were sent twice. Participants had to give online informed consent to start the checklist. Participants were asked to audit the food environment of the main location of their hospital or long-term care facility. Participants had to complete the checklist online via a tablet or laptop so they could walk around in the hospital or long-term care facility (e.g., visit the restaurants, kitchen). It was instructed to only complete one checklist per institution during a weekday and peak time of that day, assuming that most of the available food items were displayed. It should be noted that only fully completed checklists are included in the analysis.

#### Participant and health care organization characteristics

Participants of 28 hospitals and 36 long-term care facilities responded to the checklist, including 7 nursing homes, 8 rehabilitation centers, 9 institutions for people with intellectual disabilities, 11 mental healthcare institutions and 1 institution was a combination of a nursing home and rehabilitation center, as detailed in Table 3. The checklist was predominantly completed by facility staff in both hospitals (64.3%) and long-term care facilities (55.6%), followed in hospitals by policy, quality and management staff (25.0%) and in long-term care facilities by health workforce (22.2%).

**Table 3:** Characteristics of hospitals and long-term care facilities of sub-study 2 (quantitative checklist)

Total healthcare organizations (n = 64)	n (%)
**Hospitals total**	28 (100)
General	18 (64.3)
Specialized	1 (3.6)
Academic	6 (21.4)
Top-clinical	3 (10.7)
**Long-term care facilities total**	36 (100)
Mental healthcare institutions	11 (30.6)
Rehabilitation centers	8 (22.2)
For people with intellectual disabilities	9 (25.0)
Nursing homes	7 (19.4)
Combination of two or more	1 (2.8)
**Capacity for # health care receivers**	**Min-Max (Median)**
Hospitals	120–980 (405)
Long-term care facilities	4-658 (70)
**Number of employees**	
Hospitals	240-15550 (3050)
Long-term care facilities	5-2000 (150)
**Function of respondent**	**n (%)**
**Hospitals**	
Facility staff (e.g., manager food, head of hotel services, projectleader nutrition)	18 (64.3)
Health workforce (nurse, assistant, teammanager, lifestyle coach)	0 (0.0)
Dietitian	1 (3.6)
Policy, quality, management staff	7 (25.0)
Other (e.g. chef, intern, unknown)	2 (7.1)
**Long-term care facilities**	
Facility staff (e.g., manager food, head of hotel services, projectleader nutrition)	20 (55.6)
Health workforce (nurse, assistant, teammanager, lifestyle coach)	8 (22.2)
Dietitian	4 (11.1)
Policy, quality, management staff	1 (2.8)
Other (e.g. chef, intern, unknown)	3 (8.3)

#### Measures

In sub study 2 three dimensions of the food environment were assessed, the physical, political and economic dimension. The checklist audited these dimensions of the food environment via several sections: (1) general characteristics of the hospital or long-term care facility (including type of care, number of employees); (2) physical food environment characteristics (for example asking which type of food outlets were accessible, e.g. restaurant, vending machine, and which food products were served or sold); (3) political food environment characteristics (for example asking if there is a policy on food within the healthcare organization and if national dietary guidelines were applied) and 4) economic food environment characteristics (including asking the way food services and facilities were managed, in-house or outsourced with or without a profit motive). The checklist was partly inspired on the Hospital Nutrition Environment Scan for Cafeterias, Vending Machines and Gift Shops [21] and included inquiries about the food environment for health care receivers, staff and visitors. If more than one visitor- or staff restaurant was present, participants were asked to audit only the largest restaurant with the greatest variety of food and drinks available. The checklist was pilot tested by the first author (JJW) in consultation with a hospital dietitian. Based on this pilot, minor changes were made in the formulation of some food items. Due to expected variations in the food environment, the checklist for hospitals and long-term care facilities exhibited slight differences.

#### Data analysis

Descriptive statistics were used to outline general characteristics of the hospitals and long-term care facilities, and also to describe physical, political and economic characteristics of the food environment. Results were tabulated by healthcare setting type, hospital and long-term care facilities, and by food outlet type or inpatient food service. Analyses were conducted using IBM SPSS Statistics Version 28.0.

## Results

Results of both sub study 1 and 2 will be discussed per dimension of the food environment.

### Physical food environment

Semi-structured interviews indicated that in most hospitals and long-term care facilities, health care receivers were offered three meals a day, including breakfast, lunch and dinner, and a snack in-between meals. Preparation of meals for health care receivers varied between and within organizations from cook-chill- or freeze systems (rapid chilling or freezing of cooked food), regeneration (reheating food when serving) to freshly prepared (and immediately served) meals in kitchens or restaurants. Hospitals, and primarily the larger long-term care facilities used a central space for meal preparation and distribution. Predominantly, preprepared meals were delivered by external suppliers, assembled on trays and transported either to a smaller kitchen for final preparation or directly to the health care receivers. In hospitals and larger long-term care facilities, this was often done by qualified kitchen-, facility staff or nutrition assistants. Only a few long-term care facilities used an external supplier to deliver preprepared meals for their health care receivers. In smaller long-term care facilities food and drinks were often prepared in a kitchen per community room. These community-rooms served as household-like settings where health care providers or hostesses were responsible for cooking in addition to their caregiving duties.

All participants highlighted the importance of quality, taste and appearance of food and drinks, otherwise health care receivers, staff and visitors would not consume it. They argued specifically for health care receivers that eating anything at all is sometimes more important than eating something healthy. The majority of participants considered freshly cooked meals and fresh foods as the best option for their health care receivers as they were convinced that these are healthier and tastier. Moreover, freshly cooked meals elevate the ambiance in a healthcare setting and provides more opportunity for tailoring to individual preferences. Participants of a few healthcare institutions even mentioned that they had a garden to grow vegetables and fruit, where health care receivers gardened as daytime activity, ‘*They maintain the garden. It’s super fun, you can use products from your own garden for dinner*’ *(P17, team leader client services, institution for people with intellectual disabilities).* However, preparing and providing freshly cooked meals was not always feasible to do so. Participants pointed out various physical environment factors, such as the availability of facilities, logistical limitations, and the physical space of hospitals or long-term care facilities, which affected the range of methods used for their meals. For instance, a participant from a healthcare organization with multiple locations highlighted these influencing factors: *‘In the larger locations we cook for 100% convenience meals, so only regenerating meals. But we also have locations where meals are freshly cooked for 100%.’, (P16, hospitality manager, institution for people with intellectual disabilities).*

Hospitals and long-term care facilities that have on-site restaurants accessible for health care receivers, generally offered a larger food assortment and provided more variety, thereby increasing options and freedom of choice compared to long-term care facilities operating as households, where often a single meal was prepared. Only a minority of the participants mentioned that the health care receivers independently purchased and prepared their own food and drinks, for example ‘*A large part of our health care receivers can walk outside and can visit a cafeteria and so on. It is not entirely in our hands.’, (P38, coordinator services, mental healthcare institution)*. In hospitals staff often ate in the restaurant or canteen, buying something or bringing their own food and drinks from home. In long-term care facilities staff mostly ate together with health care receivers, sometimes as part of therapy. In hospitals and long-term care facilities the places where food and drinks were sold were often targeted at visitors and visitors were occasionally allowed to eat together as relative of a health care receiver.

Participants noted recent developments to move towards making healthy and sustainable foods more accessible and available, particularly in some hospitals that were affiliated with the Nutrition & Healthcare Alliance and their program initiative to realize a healthy hospital food environment in the Netherlands. Both hospitals and long-term care facilities implemented several changes in their food offerings. These changes involved providing a greater variety of whole grain products and vegetarian options while reducing the frequency of serving soft drinks and fruit juices. Additionally, they minimized the availability of fried snacks and opted to offer snacking fruit as alternatives to sugary treats.

The results of sub study 2 showed that both hospitals and long-term care facilities reported presence of different food and drink facilities, for example an on-site restaurant accessible for everyone or a restaurant for staff only and/or a coffee-lunch corner. Restaurants for staff only were less often present at long-term care facilities (13.9%) compared to hospitals (64.3%). Also, a kiosk or small gift shop selling foods was present in most of the hospitals (89.3%) and less present in long-term care facilities (25.0%). Vending machines were almost only reported in hospitals, predominantly selling a combination of soft drinks and snacks (67.9%). Vending machines selling only healthy items were the second most common type of vending machines in hospitals (42.9%). Most of all hospitals (82.1%) and long-term care facilities (91.7%) had a kitchen to fully or partly prepare food for health care receivers, Table 4.

**Table 4:** Characteristics of the food environment dimensions assessed via the checklist in sub-study 2

	Total n (%) total n = 64	Hospitals n (%) total n = 28	Long-term care facilities n (%) total n = 36
**PHYSICAL DIMENSION**			
**Type of on-site food and drink facility**			
Restaurant accessible for everyone	47 (73.4)	26 (92.9)	21 (58.3)
Restaurant for staff only	23 (35.9)	18 (64.3)	5 (13.9)
Coffee-/lunch corner	36 (56.3)	22 (78.6)	14 (38.9)
Kiosk or small (gift) shop	34 (53.1)	25 (89.3)	9 (25.0)
Supermarket	7 (10.9)	2 (7.1)	5 (13.9)
**Vending machines**			
Snacks and soft drinks combined	20 (31.3)	19 (67.9)	1 (2.8)
Soft drinks	12 (18.8)	7 (25.0)	5 (13.9)
Snacks	5 (7.8)	4 (14.3)	1 (2.8)
Healthy items	13 (20.3)	12 (42.9)	1 (2.8)
**On-site kitchen for health care receivers is present**	56 (87.5)	23 (82.1)	33 (91.7)
**The food for health care receivers is (partly) freshly cooked in on-site kitchen**	37 (57.8), 10 (15.6) partly	15 (53.6)	22 (61.1), 10 (27.8) partly
**POLITICAL DIMENSION**			
**Familiarity with national guidelines for healthy food environments (yes)**	51 (79.7)	27 (96.4)	24 (66.7)
**National guidelines for healthy food environments are (partly) applied for**			
Staff facilities (yes) Visitor facilities (yes)	47 (73.4) 40 (62.5)	25 (89.3) 23 (82.1)	22 (61.1) 17 (47.2)
**Food and drinks for health care receivers is based on national dietary guidelines (yes)**	47 (73.4)	20 (71.4)	27 (75.0)
**Developed food vision is based on national dietary guidelines for (yes)**			
Health care receivers Staff Visitors	47 (73.4) 34 (53.1) 29 (45.3)	23 (82.1) 22 (78.6) 20 (71.4)	24 (66.7) 12 (33.3) 9 (25.0)
**Food policy documents are developed and administered by interdisciplinary team (yes)**	36 (56.3)	21 (75.0)	15 (41.7)
**ECONOMIC DIMENSION**			
**Restaurant for everyone accessible (health care receivers, staff, visitors)**			
Yes, available	47 (73.4)	26 (92.9)	21 (58.3)
If yes, how is it managed?			
Outsourced In-house, profit motive In-house, no profit motive *Other*	10 (21.3) 12 (25.5) 22 (46.8) 3 (6.4)	9 (34.6) 9 (34.6) 7 (26.9) 1 (3.8)	1 (4.8) 3 (14.3) 15 (71.4) 2 (9.5)
**Restaurant only for staff**			
Yes, available	23 (35.9)	18 (64.3)	5 (13.9)
If yes, how is it managed?			
Outsourced In-house, profit motive In-house, no profit motive *Other*	2 (8.7) 3 (13.0) 17 (73.9) 1 (4.3)	2 (11.1) 2 (11.1) 14 (77.8) 0 (0.0)	0 (0.0) 1 (20.0) 3 (60.0) 1 (20.0)
**Food-service for health care receivers**			
Outsourced In-house Other	5 (7.8) 56 (87.5) 3 (4.7)	3 (10.7) 25 (89.3) 0 (0.0)	2 (5.6) 31 (86.1) 3 (8.3)

An overview of the food products offered in different food outlets in hospitals and long-term care facilities can be found in Additional file 2, Table 1. To illustrate, sugar-sweetened beverages and fruits were available in almost all food outlets in hospitals and long-term care facilities. Vegetables were offered less and plant-based beverages were present in less than half of the food outlets in hospitals and long-term care facilities. Fried snacks were offered most in hospitals with the highest percentages in restaurants for staff only (94.4%). In Table 2 of Additional file 2 food products offered via the food service for inpatients in hospitals and long-term care facilities can be found. All hospitals and long-term care facilities offer brown bread and whole meal bread for breakfast and lunch and white bread wass offered less. All hospitals offered fruit for breakfast and lunch. This was the case for 83.3% of the long-term care facilities. Vegetables were less often offered during breakfast and lunch (78.6% of the hospitals and 52.8% of the long-term care facilities). Long-term care facilities offered more unhealthy snacks like cake and pastries (47.2% vs. 28.6%) and fried snacks (47.2% vs. 25.0%) in-between meals. In hospitals fruits (96.4% vs. 88.9) and vegetables (64.3 vs. 41.7%) were more often available as a snack in between meals compared to long-term care facilities.

### Socio-cultural food environment 1

Interviews revealed that type of healthcare provided (e.g., short-term post-surgical care vs. long-term mental health care) shaped the socio-cultural food environment in healthcare settings. Participants representing hospitals highlighted that nutrition should contribute to recovery, pre-habilitation and prevention and that compliance to protein requirements was essential. This aligns with the viewpoints shared by participants representing rehabilitation centers, who further highlighted that nutrition and eating was frequently part of the health care receivers’ treatment. Participants from institutions for people with intellectual disabilities emphasized the utmost importance to engage health care receivers in the entire meal preparation process. In mental healthcare, establishing a structured rhythm for eating moments was deemed crucial. Setting limits, including those related to caffeine consumption, was considered an essential aspect of this setting. In nursing homes, most important was that food and drinks were tasty to ensure that people would eat sufficiently, illustrated by: *‘We believe it’s important to establish an environment that encourages all residents to enjoy their meals. We pay especially attention to what they are accustomed to eat at home, and ensure that there are delicious options for everyone.’, (P45, facility manager, nursing home).*

Cultural food practices not aligned with healthy eating were prevalent in both hospitals and long-term care facilities. For example, participants representing hospitals mentioned that health care receivers often used unhealthy food as a reward or to celebrate (un)favorable outcomes: ‘*Health care receivers tell us that it is nice to release tension with a cup of coffee and a sausage roll in the restaurant when they had an unpleasant doctor’s appointment*.’, *(P10, head of dietitians’ department, hospital).* Examples of similar practices were mentioned for staff, including the tradition of serving cake during birthday celebrations or offering fried snacks to commemorate a doctor’s first surgery. Regarding the food provided to health care receivers, both hospitals and long-term care facilities were consistently willing to accommodate dietary requirements and respect cultural or religious preferences related to food and drinks.

Participants from hospitals emphasized their role as model for healthy eating. They expressed the desire to set an example for health care receivers, staff and visitors, and thereby promote healthy eating practices. Advocating this exemplary role was less advocated by participants from long-term care facilities. They described their exemplary role when eating together with the health care receivers and mainly mentioned that food should be tasty and appealing. Considering that health care receivers often stay for a longer period of time it is important that food and drinks cater to their preferences. Participants from hospitals and long-term care facilities all emphasized that the care and treatment of health care receivers always took precedence. However, they noted that food did not always have an explicit role in the care process, primarily due to a lack awareness regarding the added value of healthy food in healthcare.

While it emerged from the interviews that nutrition became increasingly important within the healthcare setting, participants highlighted that health care receivers, staff and visitors do not wish to be patronized when it comes to healthy eating. To illustrate, participants mentioned that staff and visitors in hospitals showed resistance when their preferred foods were no longer available. However, health care receivers seemed to take changes more for granted. Participants indicated that most important was to stimulate healthy eating by empowering health care receivers, staff and visitors and make it more attractive instead of to discourage unhealthy eating. One participant voiced a contrasting view, suggesting that the temptation of unhealthy foods should be entirely eliminated and not be served or sold, illustrated by: ‘*Just stop tempting. Then you will see that people make different choices. It’s that simple.’, (P4, manager hotel services, hospital)*.

### Political food environment

Outcomes of the semi-structured interviews showed that all hospitals and most of the other long-term care facilities had a written document consisting of rules, goals and values concerning the food provision in the organization, often referred to as a food policy, a food vision or annual plan. Terms were interchangeably used and in this article we will refer to ‘food policy’ as term for these different designations.

During the interviews participants indicated that the support from the director- or management level for the food policy played a pivotal role in the success of both the implementation phase of food policies and in already established food policies improving food provision. Such support significantly increased the value placed on healthy eating within the organization. In addition, participants explained that a clear food policy document for the entire hospital or long-term care facility is particularly helpful in providing guidance in realizing and regulating a healthy and sustainable food environment. According to most participants, having a food policy is crucial, but its successful implementation and receiving broad organizational support were equally important for the policy to operate effectively. Participants recognized that fostering support for the food policy throughout the entire organization was a continuing process. In addition, almost all participants working at hospitals and long-term care facilities stated that the success of the implementation depends on individuals who put it into practice: *‘It shouldn’t be something top-down, the staff must be our ambassadors and transfer knowledge and skills’, (P6, catering coordinator, hospital)*. For example, while most of the hospitals and long-term care facilities actively communicated their food policy to staff and explained ‘the why’, a minority stretched the opposite and argued that implementing a food policy without publicity helped avoiding resistance. Finally, the majority of participants representing the larger long-term care facilities mentioned that each location is unique and has the freedom to adapt and implement the food policy to suit its specific needs.

In the development of food policies, the majority of participants mentioned that they adopted an interdisciplinary approach, aiming to gain support across all layers of the organization and representing all disciplines involved. Current available food policy documents were written with the nutrition of health care receivers being the core aim. Although staff and visitors were often not explicitly mentioned as a target group for nutrition policy, they were implicitly assumed to benefit from it, illustrated by *‘It [the vision/policy] applies to everyone who eats and drinks in house.’, (P11, head of nutrition, hospital).* Only a few participants of long-term care facilities mentioned not having a food policy because of conditions such as lack of priority in the organization, a high workload, or the deliberate decision to avoid a generic food policy to be able to fully customize the food for each health care receiver.

The content of the food policy document was predominantly centralized around the positive influence healthy food has on prevention, wellbeing, treatment, pre-habilitation and enhanced recovery of health care receivers. Illustrated by: *‘The policy is only two sheets of paper, it is very short, it is used as a point of departure and if I summarize it, the food policy states that food and drinks [provided by the hospital] should have a positive effect on the wellbeing of health care receivers and a positive contribution to treatment and recovery…’, (P3, policy advisor food and drinks, hospital).* Most of the hospitals and long-term care facilities referred to the ‘Wheel of Five’, a translation of the national dietary guidelines, as a basis for their food policy. However, it was also acknowledged that these guidelines not always suffice as these are designed for healthy people and sometimes adaptations were needed to meet specific needs of health care receivers. For example, the majority of the participants representing hospitals specifically mentioned that their food policy marked the importance of sufficient provision of proteins. This was distinctive from long-term care facilities where participants highlighted the importance of hospitality and meal ambiance in their food policies. Sustainable foods were often not explicitly mentioned in the existing documents and most participants emphasized that sustainability was predominantly embedded in their policies with respect to food waste or use of medical supplies.

Results from the checklist indicated that participants of almost all hospitals reported to be aware of the guidelines for food environments of the Netherlands Nutrition Centre (96.4%) and (partly) applied the guidelines in their hospital for staff (89.3%) and visitors (82.1%), as shown in Table 4. A lower percentage of participants of long-term care facilities reported to be aware of these guidelines (66.7%) and these were even less often applied in their organization for staff (61.1%) or visitors (47.2%). A total of 71.4% of the hospitals and 75.0% of health care organizations based the food provision for health care receivers on the Dutch dietary guidelines. In the majority of hospitals policy documents were developed and administered by an interdisciplinary team (75.0%), compared to less than half of the long-term care facilities (41.7%).

### Economic food environment

Based on the interviews, food services or facilities were managed either in-house or outsourced to external parties. Often, both forms were present under the same roof in a hospital or long-term care facility (e.g., there might be an outsourced visitors’ restaurant and a staff canteen managed in-house). For in-house food services, there was often no profit motive and the only goal was to break even. Such in-house services provided more space and freedom to hospitals and long-term care facilities to shift the assortment towards healthy foods. When outsourcing food services or facilities, participants mentioned that they had to deal with commercial interests and experienced less flexibility and autonomy in determining the types and prices of food offered. Illustrated by a participant: ‘*If you work with a caterer, the caterer must make profit. These external parties have a commercial interest, otherwise they don’t exist.*’, *(A1, head of hotel services, hospital).* Moreover, external parties are driven by profit motives and participants mentioned that most of the profit was primarily generated from the sale of unhealthy products. While participants indicated that little to no promotional offers or discounts were in place in hospitals or long-term care facilities, only one participant of a hospital specifically mentioned that an agreement was made with their external party to prohibit marketing for unhealthy food and added: ‘*And if we do something, we ensure it is a promotion for a product we support in the context of health*.’, *(P9, facility manager, hospital).*

Participants indicated that external parties, such as caterers, play a major role in shaping the food environment, and their involvement is often tied to long-term contracts. Consequently, they find themselves dependent on the possibilities and goodwill provided by these external parties in their transition towards a healthy and sustainable food environment. To keep control, participants used procurement policies as an opportunity to incorporate healthy and sustainable food and drinks into contracts (e.g., using criteria based on national dietary guidelines for foods and beverages sold or served). Illustrated by a participant of a hospital: ‘*We said during the procurement process, that the food concept should lead to faster recovery of health care receivers, so we included that as a key performance indicator’, (P4, manager hotel services, hospital)*. To keep flexibility, others used open-book contracts (based on actual costs, with more transparency) or best value procurement policies.

Participants mentioned that budget was an important factor in determining the foods provided for health care receivers. Hospitals and long-term care facilities usually received a fixed daily or yearly food-budget that can be used for food provision in care settings. Participants mentioned that the budget was most often enough, though sometimes challenging to provide healthy and sustainable meals. Participants’ estimation of the budgets fluctuated between seven to fifteen euros per day, *‘Of this amount, everything should be bought - coffee, breakfast, lunch and dinner. That’s quite challenging.’, (P16, hospitality manager, institution for people with intellectual disabilities).* Participants calculations differed, as some hospitals and long-term care facilities only take the ingredient costs into account, while others also include cleaning- and staff costs. Also in budgeting processes, healthy food provision often lacked priority and was commonly included as final balance item. Some participants mentioned that it was important to add a (positive) business case to the policy.

Based on the checklist in sub study 2 (Table 4), respondents of long-term care facilities reported more in-house management of restaurants for everyone accessible with no profit motive (71.4%) compared to hospitals (26.9%), where management of restaurants for everyone accessible was more outsourced or in-house with profit motive. Management of restaurants for staff was reported more in-house with no profit motive. Food for health care receivers was predominantly managed in-house for both hospitals (89.3%) and long-term care facilities (86.1%).

## Discussion

This study gained a comprehensive characterization of the food environment in hospitals and long-term care facilities. Substantial disparities in the different dimensions of the food environment between hospitals and long-term care facilities were observed. The physical dimension of the food environment in the healthcare setting is shaped by various factors, such as availability of facilities, logistic limitations and physical space. Hospitals adopt a more organized and structured method in managing the food environment for health care receivers. In contrast, long-term care facilities often exhibit a more individual-oriented approach and create an adaptable ‘homely’ food environment, tailored to individual requirements of health care receivers. The type of healthcare provided plays a decisive role in shaping the socio-cultural food environment and aligns with the needs of the residing target group. Hospitals place a more prominent focus on health in shaping their food environments and their main focus is to use nutrition for fast recovery, while long-term care facilities also used nutrition as an instrument, for example to structure the day. For the political dimension participants highlight the importance of food policies and broad organizational support for a transition of the food environment. Commercial interests, profit motives, contracts with external parties and strict budgets characterized the economic food environment. Despite the crucial role in fostering supportive food environments for everyone, both hospitals and long-term care facilities indicated that there was a limited focus on staff and visitors.

Given population ageing, it is expected that an increasing number of individuals will depend on healthcare services in the future, thereby also increasing the demand for extra healthcare workers [1, 22]. Therefore, it is crucial to invest in healthy and sustainable food environments for the future (e.g. adopting healthy food environment guidelines, procurement policies, adapted to the healthcare setting). This becomes even more significant within long-term care facilities, given that health care receivers often stay there for a prolonged period of time, which provides an opportunity to harness the potential of nutrition in promoting health and wellbeing. Long-term care facilities can learn from hospitals by adopting a similar emphasis on health when shaping their food environments. On the other hand, hospitals can draw valuable lessons from long-term care facilities, going beyond mere nutritional values, employing nutrition as tool, such as for structuring daily routines or for functional recovery. The differences in the food environment of hospitals and long-term care facilities as disclosed in this study should be taken into account when designing and implementing actions for realizing healthy food environments. Particularly, actions should be made distinctive and suitable for different healthcare settings.

Our findings regarding prevailing socio-cultural norms and beliefs about food may hinder the transition towards a healthy food environment, as it was observed that health care receivers, visitors and staff do not wish to be patronized when it comes to healthy eating; their preference is stimulating healthy eating rather than discouraging unhealthy eating. This reflects the long-standing perspective within the healthcare system favouring health *promotion* over health *protection* [23]. Moreover, they align with prevailing, neoliberal, societal norms that food choices are an individual responsibility and people should have a freedom of choice [24]. Such norms and beliefs are very powerful in shaping food environments and may affect resistance for change, especially if they are held by individuals in positions of power as they play a decisive role in shaping food environments of healthcare organizations [25]. The latter was also observed by prior studies, as those operating at the management level possess the capacity to influence the culture in the organization through budget allocations or support from external stakeholders [26, 27]. In recent years, there has been a noticeable change in the support for transitioning towards healthy food environments of healthcare boards and doctors in the Netherlands, exemplified by the developments such as the emergence of lifestyle medicine, and the Nutrition and Health Care Alliance. In the upcoming years, a more drastic shift is essential as highlighted the results of the current study.

While discussed separately, this study showed that factors within and between the four dimensions of the food environment were inherently interconnected, thereby influencing each other. The social-cultural dimension of the healthcare food environment often affected the political dimension, which, in turn, was often dependent on the economic dimension. Collectively, these three dimensions shaped the physical food environment in healthcare settings. To illustrate, support from director- or management levels helped to implement policy and to have support from the entire organization for improvement of the food environment. This interrelation of determinants of the healthcare food environment has also been observed in previous studies. For example Cranney et al. [26] found that to realize healthy hospital retail food environments, policy effectiveness and broad acceptance of the policy premise were some of the key mechanisms to achieve change. Others indicated that implementing healthy and sustainable food procurement policies can help to improve the healthiness and sustainability of the physical food environment [28, 29]. Collectively, these insights indicate that the dimensions of the food environment cannot be viewed in isolation and instead should be seen and studied as a system with factors and mechanisms around these four dimensions. Future research may use a systems approach to gain better understanding of this interconnectedness and underlying dynamics of the healthcare food environment, which has already been adopted for the wider food environment of particular settings (e.g., retail, neighbourhood) [30–32].

A strength of this study includes the mixed methods approach to gain insight into a comprehensive picture of the food environment. Another strength is the large diversity of hospitals and long-term care facilities included as well as the focus on all relevant target groups including health care receivers, staff and visitors. This study also has some limitations. First, (in the majority of the interviews) only one representative of each healthcare setting was interviewed and may not be representative for the viewpoints of all stakeholders of that hospital or long-term care facility. The food environment checklist was also filled in by a single staff member and for example in larger long-term care facilities the checklist was sometimes filled in for and by multiple (different) locations (e.g. daycare centers). More objective insights could have been obtained by auditing the healthcare food environments by an independent person, not related to the hospital or long-term care facility, at multiple unannounced moments or by assessing the purchase orders of the healthcare food environments. Second, quantitative data was collected at a single point, not reflecting the variability over time. Third, most of the participating hospitals were part of the Nutrition & Healthcare Alliance. This should be taken into account as this may have resulted in an overestimation of the healthiness of food environments compared to the majority of hospitals not involved with the Alliance. And last, data were collected during the COVID-19 pandemic that may have caused that the food environment differed from a normal situation. It is not expected that it affected the study because participants were asked to reason from a normal situation during interviews.

This study gives first insights where there is room for improvement in the different domains of the food environment. Recommendation for future research is to explore how to accomplish a transition of the food environment in the healthcare setting towards a healthy and sustainable food environment, incorporating different types of care. A possible way to achieve this is to study the food environment in the healthcare setting as a complex environment, using systems thinking and understand the factors and mechanisms of the physical, socio-cultural, political and economic dimensions altogether. Another future research priority might be to study what facilitates a cultural shift in beliefs and norms within the entire healthcare setting to let health care receivers, staff and visitors see the importance of healthy and sustainable eating. Establishing these beliefs and norms is needed to create support for changing the food environment and overcome resistance. Although our study originally aimed to also explore sustainability considerations in the food environment in hospitals and long-term care facilities, participants almost exclusively emphasized health considerations. This might suggest that in the healthcare setting there is less awareness of the role of food environments in planetary health but also that the health context may implicitly evoke healthy associations more than sustainability ones. Future studies may explicitly study sustainability aspects, as attaining sustainability alongside health remains vital for transition of the healthcare food environment.

## Conclusions

This study characterized the food environment in Dutch healthcare settings, and disclosed several differences between hospitals and long-term care facilities in healthiness of the food environment. For instance, whereas hospitals emphasized nutrition for fast recovery, long-term care facilities more often approached food and eating as an instrument, i.e. to structure the day. Also it was found that hospitals are currently making positive adjustments to the food environment, such as offering whole grain breads and minimizing the availability of fried snacks. Less progress was observed in long-term care facilities. Also similarities were found. For instance, both hospitals and long term-care facilities highlighted the crucial role of having a food policy and broad organizational support for food policy. For both healthcare types, commercial interests and strict budgets were identified as important factors to recognize when improving food environments. However, food services managed in-house, without profit motive, provided often more opportunities and freedom to shift the assortment towards healthier foods. To facilitate a transition towards a healthy food environment in the entire Dutch healthcare landscape, it is imperative to incorporate all healthcare settings into designing approaches for implementation of improvements. Moreover, it is important to extend the focus beyond health care receivers and encompass the food environment for staff and visitors, and attain sustainability alongside healthiness of healthcare food environments.

### Electronic supplementary material

Below is the link to the electronic supplementary material.

Supplementary Material 1

Supplementary Material 2

## Data Availability

The datasets used and/or analyzed during the current study are available from the corresponding author on reasonable request and with permission of the Nutrition & Healthcare Alliance.

## Footnotes

1 The concepts of the socio-cultural dimension of the food environment seem to be illustrated at an individual level, however, during the interviews participants were asked to describe and reflect on a general tendency in their healthcare organization.
