# Supplementary material for: Characterizing food environments of hospitals and long-term care facilities in the Netherlands: a mixed methods approach
Source: BMC Health Serv Res. 2024 Jan 4;24:31. doi: 10.1186/s12913-023-10399-6 (PMC10768251; doi:10.1186/s12913-023-10399-6)
Supplement: Supplementary file 1 — Supplementary Material 1 [file 12913_2023_10399_MOESM1_ESM.docx]

**Additional file provided**

**File name:** Additional_File_1_Wierda_et_al_Characterizing food environments of hospitals and long-term care facilities_13_10_2023

**File format including the three-letter file extension:** Microsoft Word, DOC

**Title of data:** Interview guide for semi-structured interview

**Description of data:** The full interview guide (translated from Dutch to English) can be found in Additional file 1. The interview guide was used to obtain information regarding four dimensions of the food environment in hospitals and long-term care facilities.

**Additional File 1**

**Interview guide for the semi-structured interviews with questions and prompts, used to obtain information regarding the food environment in hospitals and long-term care facilities.**

**Introduction and physical food environment**

*Thank you for participating in this interview.*

1. Since when have you been working in the hospital/long-term care facility?

2. What is your function within the hospital/long-term care facility? On which location(s) are you working?

3. The interview is about the food and drinks in the hospital/long-term care facility and not about you, but I’m curious, what do you think in general of the food and drink offer in the hospital/long-term care facility?

> Could you please provide information on how the food and drinks are organised in the hospital/long-term care facility?

> Is the offer for health care receivers, staff and visitors organised separate or together? Managed in-house or outsourced?

> Where can you get food and drinks and how do people eat?

**Socio-cultural food environment**

*Attitude*

4. How do people within the hospital/long-term care facility think about healthy eating and drinking, from the perspective of: the management board, facility management, staff, health care receivers, caterer(s)?

5. How do people within the hospital/long-term care facility think about sustainable eating and drinking, from the perspective of: the management board, facility management, staff, health care receivers, caterer(s)?

*Culture*

6. Which culture prevails in the hospital/long-term care facility when it comes to healthy eating and drinking? With culture I mean the whole of norms, values, traditions and rules of the hospital/long-term care facility.

7. Which culture prevails in the hospital/long-term care facility when it comes to sustainable eating and drinking? With culture I mean the whole of norms, values, traditions and rules of the hospital/long-term care facility.

*Extra questions for long-term care facilities – habits:* # Which habits are in place in the long-term care facility regarding healthy eating and drinking? With habit I mean a commonly accepted practice or habit. For example, every Friday fries? Ever Saturday a cake? Fish on Wednesday?

# Which habits are in place in the long-term care facility regarding sustainable eating and drinking? With habit I mean a commonly accepted practice or habit.

*Modelling*

8. To what extent does the hospital/long-term care facility want to give the right example regarding healthy eating and drinking? If yes, how and to whom? For example to health care receivers, staff, visitors, the outside world, and/or other hospitals/long-term care facilities? If not, why not?

9. To what extent does the hospital/long-term care facility want to give the right example regarding sustainable eating and drinking? If yes, how and to whom? For example to health care receivers, staff, visitors, the outside world, and/or other hospitals/long-term care facilities? If not, why not?

*Empowerment*

10. To what extent is the hospital/long-term care facility supporting her staff to eat healthy? If yes, how is the hospital/long-term care facility supporting them in this? And what about visitors? And health care receivers? If not, why not?

11. To what extent is the hospital/long-term care facility supporting her staff to eat sustainable? If yes, how is the hospital/long-term care facility supporting them in this? And what about visitors? And health care receivers? If not, why not?

12. To what extent is the hospital/long-term care facility supporting external stakeholders (caterers, suppliers, to whom the hospital/long-term care facility is outsourcing the eat- and drink facilities) to realize a healthy offer of food and drinks? If yes, how is the hospital/long-term care facility stimulating them in this? If not, why not?

13. To what extent is the hospital/long-term care facility supporting external stakeholders (caterers, suppliers, to whom the hospital/long-term care facility is outsourcing the eat- and drink facilities) to realize a sustainable offer of food and drinks? If yes, how is the hospital/long-term care facility stimulating them in this? If not, why not?

*Extra questions for long-term care facilities:* # Can you provide some information about the role staff on the floor have in the long-term care facility during eating and drinking moments? Do they stimulate/encourage health care receivers to consume healthy and sustainable food an drinks?

# To what extent are staff aware of healthy and sustainable eating? Does this have a role in their work? Do they stimulate health care receivers for example in making the health and sustainable choice?

> Are there training programs for staff to stimulate healthy and/or sustainable eating among health care receivers?

# Can you tell something about the preferences and needs of the target group in your long-term care facility, regarding eating and drinking? Do the preferences and needs play a role in determining what is consumed?

**Political food environment**

14. Does the hospital/long-term care facility have a vision regarding food and drinks in the hospital/long-term care facility, a vision on nutrition?

> If yes, what does it entails?

> If no, do you know why there is no vision on this? Do you know what could be of influence?

15. Does the hospital/long-term care facility have a policy regarding food and drinks in the hospital/long-ter care facility?

> If yes, what does the policy entails? Is the policy separated for health care receivers, staff and visitors? Where is it written down, is there a document available? Can I read the document, can you mail it?

> If no policy, do you know why there is no policy on this? Do you know what could be of influence?

16. Can you describe what is mentioned in the policy about healthy food and drinks in the hospital/long-term care facility? If nothing is mentioned, do you know the reason why?

> Is the policy separated for health care receivers, staff and visitors? If yes, what is mentioned for health care receivers? What is mentioned for staff? What is mentioned for visitors?

> To what extent and how are health care receivers, staff and visitors informed about the policy?

> With what kind of rules and/or regulations must the food and drinks offer comply, as mentioned in the policy of the hospital/long-term care facility? You can think of the Dutch dietary guidelines and the Wheel of five.

> Are there any restrictions concerning the food and drinks offered? If yes, what are those restrictions and how were those established?

> In the determination of the provision of food and drinks, is health considered as a procurement criterion (requirements and preferences in the tendering process)?

17. Can you describe what is mentioned in the policy about sustainable food and drinks in the hospital/long-term care facility? If nothing is mentioned, do you know the reason why?

If something is mentioned about sustainability:

> Is the policy separated for health care receivers, staff and visitors? If yes, what is mentioned for health care receivers? What is mentioned for staff? What is mentioned for visitors?

> To what extent and how are health care receivers, staff and visitors informed about the policy?

> In the determination of the provision of food and drinks, is sustainability considered as a procurement criterion (requirements and preferences in the tendering process)?

> How is this monitored? For instance, impact on the environment, seasonal fruits and vegetables?

> To what extent is preference given to local products/suppliers?

18. Who made the policy? Who were involved*? Could you please tell something more about who determines what the offer of food and drinks for health care receivers, staff and visitors? * (Think of integrated policy/ formulated by an interprofessional team, for example facility management, dietician, kitchen, nurse).

19. How are policies, rules and/or regulations adhered to? And if yes, how is this controlled/monitored/assessed?

**Economic food environment**

20. To what extent do profit and loss play a role in determining the food and drink offer in the hospital/long-term care facility? Are there any economic considerations whether to sell or not sell food and drinks? And how does this differ per facility, for example meals for inpatients versus the restaurant for visitors? Could you provide information which food offer generates most profit? Do you intentionally strategize your offer to this?

21. Are the healthy food and drinks more expensive compared to the unhealthy food and drinks in the hospital/long-term care facility? If yes/no, why? And how is this for sustainable- versus non sustainable food and drinks?

22. > Eat and drink facilities for visitors, staff and health care receivers: Is healthy eating and drinking promoted in for example the restaurant or coffee corner? For instance by promotions, as discount and combo deals? Is sustainable eating and drinking promoted? For instance by promotions, as discount and combo deals?

> Staff: does the hospital/long-term care facility offer any price incentives for employees who pay for food and drinks? If yes, how? (Is there a difference in the extent of the price incentive for healthy versus unhealthy food and beverages?)

> Health care receivers: what is the daily food-budget for a health care receiver who resides in the hospital/long-term care facility? And what are the daily expenses the hospital/long-term care facility has for food and drinks for a health care receiver? Is there a difference between these two (budget and expenses)? If yes, why? Is the daily-food budget sufficient to provide a complete offer of healthy and sustainable food and drinks to the health care receiver?

**Transition towards a healthy and sustainable food environment – how to realize?**

23. What is currently going well in your hospital/long-term care facility regarding the healthiness and sustainability of the food environment?

24. Where do you believe there are still opportunities/chances for improvement? What are the risks? What are barriers to realize this?

25. What are ambitions for the coming years concerning food and drinks, in terms of the offer, policies and pricing? Where does the hospital/long-term care facility aim to be and when does it intend to reach these goals? What is the current focus in this regard?

26. During this interview I have heard many stakeholders of the food environment – including caterer, supplier, dietician. Which stakeholders haven’t been mentioned today, but are important stakeholders of the food environment?

27. Are you aware of-/ what do you think of the ambition of the National Prevention Agreement, that states that all hospitals should have a completely healthy offer of food and drinks in 2030? And ensuring a healthier food offer in other types of healthcare institutions?

> If yes, what do you think of this ambition and why? Do you think it is good, or not good? Needed? Financially feasible/realistic? Organizationally feasible/realistic?

> If no, [interviewer explains the ambition]: the Dutch government, in collaboration with many other organisations, has established agreements and documented them in the National Prevention Agreement, with important steps towards achieving a healthier Netherlands, by addressing smoking, overweight, and problematic alcohol consumption. One of these agreements is that all hospitals should have a healthy food offer in 2030 (for health care receivers, visitors and staff). And ensuring a healthier food offer in other types of healthcare institutions. What do you think of this ambition? Do you think it is good, or not good? Needed? Financially feasible/realistic? Organizationally feasible/realistic?

*Only for hospitals:* If the hospital is involved as a ‘frontrunner hospital’ in the initiative of the Nutrition & Healthcare Alliance: in what way are you, as frontrunner hospital/Goede Zorg Proef Je hospital of the Nutrition & Healthcare Alliance, working to realize the ambition in an accelerated pace?

If the hospital is not a ‘frontrunner hospital’: are you aware of the Goede Zorg Proef Je/ frontrunner hospitals of the Nutrition & Healthcare Alliance? Are you involved in this initiative? If yes, how? If no, why not?

28. What is needed in your hospital according to you to realize the ambition of the National Prevention Agreement? Think for example of selling less or no more fried snacks, making the price of healthy products lower compared to unhealthy products, or a different vision of a caterer or hospital or long-term care facility.

> What do you need for this? > Who could help you with this? > What is needed from the management board, which adjustments, agreements or attitude? > What is needed from the in-house management situation? Which adjustments, agreements or attitude? > What is needed from the outsourced situation? Which adjustments, agreements or attitude? > What is needed from the consumers? For example the visitors who eat in the visitor restaurant or the staff who eat in the restaurant for staff?

**Closing**

Is there anything you want to add? Something we already discussed? Something we have not discussed yet? Do you have any remaining questions? *Thank you very much for your time and shared information.*
