# Supplementary material for: Characterizing food environments of hospitals and long-term care facilities in the Netherlands: a mixed methods approach
Source: BMC Health Serv Res. 2024 Jan 4;24:31. doi: 10.1186/s12913-023-10399-6 (PMC10768251; doi:10.1186/s12913-023-10399-6)
Supplement: Supplementary file 2 — Supplementary Material 2 [file 12913_2023_10399_MOESM2_ESM.docx]

**Additional file provided**

**File name:** Additional_File_2_Wierda_et_al_Characterizing food environments of hospitals and long-term care facilities_13_10_2023

**File format including the three-letter file extension:** Microsoft Word, DOC

**Title of data:** Food products offered in different food outlets and via the food service for inpatients

**Description of data:** An overview of the food products offered in different food outlets in hospitals and long-term care facilities can be found in Table 1 of Additional file 2. In Table 2 of Additional file 2 food products offered via the food service for inpatients in hospitals and long-term care facilities can be found.

**Additional File 2**

**Table 1** Food products offered in hospitals and long-term care facilities displayed per food outlet type

|  | **Hospitals**  **n (%)** |  | **Long-term care facilities**  **n (%)** |  |
| --- | --- | --- | --- | --- |
|  | **Restaurant for everyone**  Yes, present  = 26 (92.9%) | **Restaurant for staff only**  Yes, present  = 18 (64.3%) | **Restaurant for everyone**  Yes, present  = 21 (58.3%) | **Restaurant for staff only**  Yes, present = 5 (13.9%) |
| Croissants and  puff pastry snacks | 25 (96.2) | 8 (44.4) | 10 (47.6) | 1 (20.0) |
| Sweets and chocolates | 16 (61.5) | 6 (33.3) | 12 (57.1) | 2 (40.0) |
| Fried snacks | 23 (88.5) | 17 (94.4) | 13 (61.9) | 3 (60.0) |
| Crisps and salted savoury snacks | 13 (50.0) | 7 (38.9) | 12 (57.1) | 2 (40.0) |
| Nuts non-salted | 14 (53.8) | 9 (50.0) | 5 (23.8) | 1 (20.0) |
| Cakes and pastries | 22 (84.6) | 5 (27.8) | 12 (57.1) | 1 (20.0) |
| Biscuits, muesli bars | 23 (88.5) | 15 (83.3) | 16 (76.2) | 4 (80.0) |
| Ice cream | 13 (50.0) | 1 (5.6) | 13 (61.9) | 2 (40.0) |
| Fruits | 24 (92.3) | 18 (100.0) | 20 (95.2) | 4 (80.0) |
| Vegetables | 16 (61.5) | 15 (83.3) | 12 (57.1) | 2 (40.0) |
| Free water | 17 (65.4) | 18 (100.0) | 19 (90.5) | 4 (80.0) |
| Paid water | 26 (100.0) | 18 (100.0) | 15 (71.4) | 3 (60.0) |
| Sugar-Sweetened Beverages | 24 (92.3) | 16 (88.9) | 18 (85.7) | 3 (60.0) |
| Sugar free beverages (diet or light) | 26 (100.0) | 17 (94.4) | 18 (85.7) | 3 (60.0) |
| Fruit juices (freshly squeezed) and smoothies | 22 (84.6) | 15 (83.3) | 12 (57.1) | 3 (60.0) |
| Skimmed milk, semi-skimmed milk and buttermilk | 25 (96.2) | 18 (100.0) | 21 (100.0) | 5 (100.0) |
| Whole milk | 2 (7.7) | 0 (0.0) | 5 (23.8) | 0 (0.0) |
| Plant based beverages (dairy substitutes) | 11 (42.3) | 6 (33.3) | 10 (47.6) | 1 (20.0) |
| Sweetened dairy drinks | 21 (80.8) | 18 (100.0) | 16 (76.2) | 5 (100.0) |
| Brown bread and wholemeal bread | 23 (88.5) | 18 (100.0) | 21 (100.0) | 5 (100.0) |
| White bread | 21 (80.8) | 15 (83.3) | 16 (76.2) | 5 (100.0) |
| Cold meat cuts | 16 (61.5) | 18 (100.0) | 20 (95.2) | 5 (100.0) |
| Cold meat cuts substitutes (vegetarian) | 8 (30.8) | 8 (44.4) | 6 (28.6) | 3 (60.0) |
| Low-fat cheese | 13 (50.0) | 16 (88.9) | 15 (71.4) | 4 (80.0) |
| Full fat cheese | 16 (61.5) | 18 (100.0) | 17 (81.0) | 5 (100.0) |
| Salad spreads for bread | 15 (57.7) | 18 (100.0) | 14 (66.7) | 4 (80.0) |
| Other savoury vegetarian bread filling | 12 (46.2) | 16 (88.9) | 14 (66.7) | 3 (60.0) |
| Sweet bread filling | 16 (61.5) | 18 (100.0) | 17 (81.0) | 5 (100.0) |

**Table 2** Food products offered to health care receivers in hospitals and long-term care facilities

|  | **Hospitals**  **n (%), n total = 28** | **Long-term care facilities**  **n (%), n total = 36** |
| --- | --- | --- |
| **Products offered for breakfast and lunch** |  |  |
| Fruits | 28 (100.0) | 30 (83.3) |
| Vegetables | 22 (78.6) | 19 (52.8) |
| Water | 28 (100.0) | 30 (83.3) |
| Sugar-Sweetened Beverages | 26 (92.9) | 16 (44.4) |
| Sugar free beverages (diet or light) | 19 (67.9) | 11 (30.6) |
| Fruit juices (freshly squeezed) and smoothies | 12 (42.9) | 15 (41.7) |
| Skimmed milk, semi-skimmed milk and buttermilk | 28 (100.0) | 36 (100.0) |
| Whole milk | 15 (53.6) | 11 (30.6) |
| Plant based beverages (dairy substitutes) | 15 (53.6) | 17 (47.2) |
| Sweetened dairy drinks | 17 (60.7) | 16 (44.4) |
| Brown bread and wholemeal bread | 28 (100.0) | 36 (100.0) |
| White bread | 26 (92.9) | 23 (63.9) |
| Cold meat cuts | 28 (100.0) | 36 (100.0) |
| Cold meat cuts substitutes (vegetarian) | 14 (50.0) | 17 (47.2) |
| Low-fat cheese | 26 (92.9) | 28 (77.8) |
| Full fat cheese | 25 (89.3) | 28 (77.8) |
| Salad spreads for bread | 22 (78.6) | 14 (38.9) |
| Other savoury vegetarian bread filling | 24 (85.7) | 29 (80.6) |
| Sweet bread filling | 27 (96.4) | 35 (97.2) |
| **Products offered as snacks in-between meals** |  |  |
| Croissants and  puff pastry snacks | 6 (21.4) | 16 (44.4) |
| Sweets and chocolates | 4 (14.3) | 13 (36.1) |
| Fried snacks | 7 (25.0) | 17 (47.2) |
| Crisps and salted savoury snacks | 5 (17.9) | 16 (44.4) |
| Nuts non-salted | 18 (64.3) | 15 (41.7) |
| Cakes and pastries | 8 (28.6) | 17 (47.2) |
| Biscuits, muesli bars | 21 (75.0) | 27 (75.0) |
| Ice cream | 15 (53.6) | 11 (30.6) |
| Fruits | 27 (96.4) | 32 (88.9) |
| Vegetables | 18 (64.3) | 15 (41.7) |
